# Spinose ear tick (Otobius megnini) infestation: Parasite burden and milk production response following permethrin aerosol in-ear treatment in a California dairy herd

**DOI:** 10.3168/jdsc.2026-1020

**Published:** 2026-05-01

**Authors:** P. Jardon, A. Rico, A. Gerry, B. Karle, N. Silva-del-Rio

**Affiliations:** 1 College of Veterinary Medicine, Iowa State University, Ames, IA 50011; 2 Department of Population Health and Reproduction, UC Davis School of Veterinary Medicine, Davis, CA 95616; 3 UC Davis Veterinary Medicine Teaching and Research Center, Tulare, CA 93274; 4 Department of Entomology, University of California Riverside, Riverside, CA 92521; 5 Cooperative Extension, Division of Agriculture and Natural Resources, University of California, Orland, CA 95963

## Abstract

The spinose ear tick (*Otobius megnini*) is an ectoparasite of cattle whose impact on dairy production is poorly defined. This study evaluated the efficacy of a permethrin-based control protocol on tick burden and milk yield in Holstein cows under commercial California dairy conditions. Midlactation cows (n = 647) were systematically allocated to treatment groups by ear tag (odd or even) to receive either no treatment (CON; n = 300) or topical applications of permethrin aerosol spray (0.5%; ∼3 mL per ear; a 296-mL can treated approximately 50 cows) at wk 0 and 2 (TT; n = 347). Tick scores (TSc; 0 = none; 1 = 1–5; 2 = 6–10; 3 = >10 ticks/ear) were assessed at baseline and at wk 2 and 3. Weekly milk yield was derived from automated parlor records for −7 to 28 d relative to treatment. Cumulative logit models were used to evaluate changes of TSc over time, and linear mix models adjusted for milk yield at baseline and pen were used to evaluate weekly milk-yield data. A significant reduction in TSc was observed for TT compared with CON, with a significant treatment × week interaction. The predicted probability of TSc = 0 (no ticks/ear) at wk 3 postintervention was 0.1% (95% CI: 0.0–0.2) for CON and 83.4% (95% CI: 75.9–88.9) for TT versus 0.3% and 1.2%, respectively at wk 0. After adjusting for baseline milk yield and pen, no treatment effect on weekly milk yield was observed: 39.7 versus 39.6 kg/d for CON and TT, respectively. Results demonstrated that permethrin aerosol effectively reduced spinose ear tick infestation but did not improve short-term milk production. This study provides field-based evidence that permethrin can substantially reduce tick burden in US dairy cattle and calls for longer-term studies evaluating production, welfare, and reinfestation outcomes.

## Highlights

• Permethrin spray (0.5%) applied twice reduced spinose ear tick burden in Holstein cows.
• Under commercial conditions, short-term milk yield was not affected by treatment.

## Graphical Abstract

**Figure:**
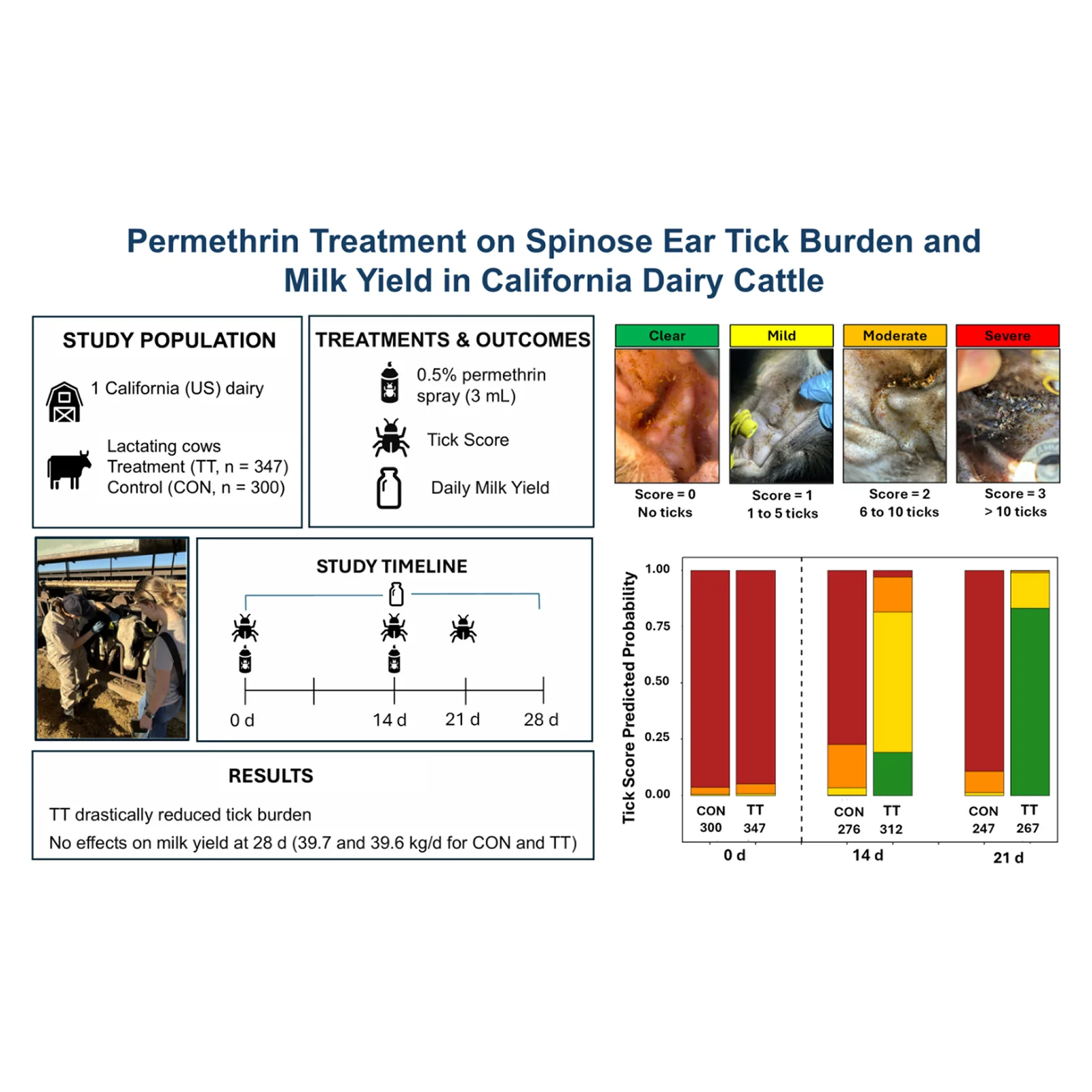
**Summary:** Spinose ear ticks are common parasites in dairy cattle, but their effects on milk production are unclear. In this study, more than 600 Holstein cows on a California dairy were either untreated (CON) or given 2 applications of 0.5% permethrin spray in the ears. Treated cows (TT) had significantly fewer ticks, but milk production did not change over the 28-day study period compared with untreated cows. These results show that 0.5% permethrin spray is effective at reducing tick burden in the ears of cattle, although further research is needed to understand long-term effects on production, animal welfare, and reinfestation.

The spinose ear tick (*Otobius megnini*) is a one-host soft tick (Family *Argasidae*) whose larval and nymphal stages parasitize the ears of ungulates, particularly cattle, horses, and other livestock species. Although primarily found in the ear canal, ticks have also been reported under the tails of infested cows (Bulman and Walker, 1979). Occasional infestations have been documented in wildlife species (e.g., deer, antelope) and in humans with close contact with infested animals (Rich, 1957; Naudé et al., 2001). In cattle, heavy infestations can cause intense irritation, persistent head shaking, and restlessness, potentially leading to otic lesions, secondary bacterial infections, and screwworm myiasis in endemic areas (Parish, 1942; Bulman and Walker, 1979).

Although these clinical signs raise animal welfare concerns, the economic impact of spinose ear tick infestations in dairy cattle remains poorly defined. Early literature provided anecdotal observations suggesting reduced milk yield, increased weight loss, and even death in affected animals, but controlled studies were lacking (Rich, 1957; Strickland et al., 1976; Bulman and Walker, 1979). To date, no controlled field studies have assessed the production effects of spinose ear tick infestation under commercial US dairy conditions.

Originally native to the southwestern region of North America (Dugès, 1883, Strickland et al., 1976), the spinose ear tick is now widely distributed across the Americas, from southern Canada to South America, and has been introduced to distant regions, including Hawaii, Africa, Sri Lanka, and India, through the movement of infested animals (Bishopp and Trembley, 1945; Keirans and Pound, 2003; Rajakaruna and Diyes, 2019). Although the presence in commercial dairy operations in California has been recently confirmed (Jardon et al., 2018), archival evidence documents the tick in California dairy cattle at least as early as the 1940s (Jellison et al., 1948).

Uniquely among ticks, only the larval and nymphal stages of spinose ear ticks are parasitic. These immature stages reside within the host's ear canal for extended periods (up to 7 mo), while completing development (Kemper and Peterson, 1953; Herrero, 1989). Adult females can deposit multiple small egg batches in sheltered environments such as cattle bedding or resting areas, where larvae hatching from these eggs await hosts (Herms, 1950; Niebuhr et al., 2013).

Historically, tick control relied on topical treatments such as pine tar, petroleum distillates, and organophosphate sprays (e.g., chlorpyrifos, coumaphos), which offered rapid control of infestation but limited residual activity (Kemper and Peterson, 1953; Drummond et al., 1967). More recently, synthetic pyrethroids such as permethrin have been used in ear tags and topical sprays (Lancaster, 1984). However, peer-reviewed data on the efficacy of these treatments and their potential influence on milk yield in commercial settings remains limited.

The objective of this study was to evaluate the efficacy of a 0.5% permethrin (Insecticide Resistance Action Committee Group 3A) aerosol spray, administered as 2 topical applications 15 d apart in the ears of dairy cows, in spinose ear tick infestation and milk production in mid-lactation Holstein cows under commercial dairy conditions.

All procedures in this study were approved by the Institutional Care and Use Committee at Iowa State University (IACUC-24-142-California Spinose Ear Tick Study).

The study was conducted on a commercial dairy in Northern California (United States) from October to November 2024. The farm was selected based on its documented presence of spinose ear tick, availability of electronic daily milk-yield records (Beco Dairy Automation Inc., Hanford, CA), well-established management practices, and willingness to participate in the study.

The dairy operation housed 2,336 lactating Holstein cows in freestall barns bedded with dried manure solids. Cows were distributed across 9 onsite lactating pens and 2 dry cow pens at a nearby offsite location. Stalls were groomed twice daily, and fresh bedding was added once weekly. The farm employed heat abatement measures, including fans and soakers. Routine health management included multiple daily fresh cow pen walk-through, daily milk weight evaluation by trained staff, monthly herd veterinarian visits, and adherence to veterinary prescribed vaccination protocols.

Cows were milked twice daily using a 60-cow rotary parlor. They were fed a TMR once per day, formulated to meet the nutritional requirements of lactating cows. Estrus detection relied primarily on tailhead chalk, with a subset of animals enrolled in a timed artificial insemination program. The herd had an average 305-d mature equivalent milk yield of 13,235 kg/cow. Although all female replacement calves were raised on site, the herd was not fully closed; occasional purchases of animals from outside sources occurred.

A longitudinal study was conducted in mid-lactation cows housed in 2 separate pens. These pens were selected based on the dairy's ability to maintain relatively stable groups throughout the study period, such that cow movement to other pens during the experiment would be minimized. Because heavy spinose ear tick infestation was observed across all lactating pens, these pens were considered representative of the lactating herd in terms of infestation status. The cows were systematically allocated within each pen to one of 2 treatments; cows with even numbered herd identification (**ID**) tags were assigned to control (**CON**; no treatment, n = 300) and cows with odd numbered ID tags were assigned to tick treatment (**TT**; treatment, n = 347). Cows assigned to the TT group received one application (∼3 mL) of a 0.50% permethrin aerosol spray (Catron IV, Elanco Animal Health, Greenfield, IN) applied inside the ear canal at study initiation and again 15 d later. The product label instructions for spinose ear tick control (“Spray downward directly into animal's ears”) were followed. Ticks were thoroughly soaked with spray, with an application time of approximately 1 s per ear, although this varied somewhat depending on cow temperament. A 296-mL can treat approximately 50 cows, corresponding to an estimated application volume of about 3 mL per ear. No adverse reactions were observed other than an immediate startle response to the sound and sensation of the spray and brief head shaking lasting a few seconds in almost all treated cows. Because the product was approved with no milk or slaughter withdrawal time, no milk or meat withdrawal periods were observed.

Because of the lack of prior data on the effects of spinose ear tick infestation on milk yield in dairy cattle, sample-size calculations were based on detecting a meaningful minimum difference of 3 kg/d in milk yield between CON and TT groups. A sample size of 179 cows per group was deemed necessary to detect a 3-kg difference in milk yield at the 5% significance level with 80% power, using an SD of 9.5 kg/d based on prior studies from our laboratory (García-Muñoz et al., 2017). The number of cows included in the study was above the sample-size calculation to account for loss of cows to follow-up due to standard herd management practices such as dry-off, culling, or pen movement.

Tick infestation was assessed in both ears at baseline (wk 0, pretreatment) and again at 2 and 3 wk post-treatment. To establish and validate the tick scoring method, 3 members of the research team (AG, PJ, and BK) conducted preliminary training on tick counts using cows from the same facility before study initiation. For the trial itself, all tick assessments were conducted by a single researcher (BK) to eliminate observer bias. During each tick scoring event, the observer used a pen light to facilitate observation of ticks within the ear canal of the restrained cow. Complete blinding was not possible because the ID tag was located in the ear being evaluated. Although evaluators attempted to remain unaware of animal identity during scoring, this was not always feasible. Both ears were examined on all cows, with the tick score (**TSc**) for the animal recorded as the highest TSc observed in either ear. Cows were classified as score 0 (0 ticks/ear), score 1 (1–5 ticks/ear), score 2 (6–10 ticks/ear), or score 3 (>10 ticks/ear) as shown in the Graphical Abstract. This TSc methodology was designed based on the research team expertise and the observed prevalence of ticks at this dairy before study initiation.

Daily milk-yield data, obtained from the dairy management software (DHI-Plus, Amelicor, Provo, UT) was recorded for 4 wk following treatment, and weekly averages were calculated to assess changes over time. Daily milk-yield data were collected from the Beco management software from −7 to 28 d relative to date of initial treatment.

Significance was set at *P* < 0.05. Before model fitting, we assessed model assumptions using visual residual diagnostics, including a normal quantile-quantile plot of residuals and residuals versus fitted values. At enrollment, differences between CON and TT groups were assessed using Student's *t*-tests for continuous variables (milk yield, DIM) and Chi-squared tests for categorical variables (parity, pen, and TSc). The same approach was applied to compare cows lost to follow-up between groups.

Tick scores were modeled using a cumulative logit mixed-effects model (ordinal package in R; Christensen, 2023). Fixed effects included treatment, week, treatment × week, and pen. Week was analyzed as a repeated measure with cow-within-treatment as a random intercept. The treatment × week interaction was evaluated using a likelihood ratio test (**LRT**) by comparing models with and without the interaction term. Predicted probabilities of TSc were estimated for all combinations of treatment and week (with pen set at its reference level), following the approach described by García-Muñoz et al. (2017). Predicted probabilities for each TSc category (defined at enrollment) were computed manually using the model's estimates, following the proportional odds logistic regression framework (Fagerland and Hosmer, 2013). Linear predictors were derived for each combination of treatment and week. The TSc-specific probabilities were calculated from the cumulative logits using inverse-logit transformations. Model fit was evaluated by comparing the final model with the null model using both the LRT and the Akaike information criterion (**AIC**).

Milk yield was analyzed using a linear mixed-effects model (nlme package in R; Pinheiro and Bates, 2025). Fixed effects included treatment, week, treatment × week, pen, and baseline milk yield (1-wk average milk yield measured before treatment), which was included as an uncentered covariate. Week was modeled as a repeated measure with cow-nested-within-treatment as the subject. An autoregressive covariance structure was selected based on the lowest AIC. The interaction between treatment and week was tested using LRT comparing models with and without the interaction term. Residuals of the final model were examined to assess normality and homoscedasticity. Least squares means and SE were reported (emeans package; Lenth, 2025). Goodness of fit was assessed by comparing the final model with the null model using the LRT and the AIC.

Cow characteristics at enrollment are presented in Table 1. At enrollment, no significant differences were detected between CON and TT in parity (*P* = 0.97), weekly milk yield (*P* = 0.15), days in milk (*P* = 0.12), pen (*P* = 0.58), or TSc (*P* = 0.39). During the study, some cows were lost to follow-up because of management decisions (CON = 62; TT = 87). Among these cows, no group differences were observed in parity (*P* = 0.61), weekly milk yield (*P* = 0.30), DIM (*P* = 0.21), pen (*P* = 1.00), or TSc (*P* = 0.12).

**Table 1:** Baseline characteristics of cows at enrollment assigned to either the control group (CON) or the treatment group (TT; ˜3 mL of 0.50% permethrin aerosol spray in the ear at 0 and 2 wk) for spinose ear tick

Item	CON	TT	*P*-value
Cows (n)	300	347	
Milk yield (kg/d)	38.6	37.9	0.15
DIM	161	171	0.12
Parity (%)			0.97
1	43.7	45.8	
2	22.7	21.6	
3	15.7	15	
>3	17.9	17.6	
Pen (%)			0.58
A	39.3	41.8	
B	60.7	58.2	
Tick score (%)			0.39
0 (no ticks)	0.3	1.2	
1 (1–5 ticks)	7	6.6	
2 (6–10 ticks)	18.7	23.6	
3 (>10 ticks)	74	68.6	

There was a significant reduction of TSc for TT versus CON, with a significant treatment × week interaction for TSc (*P* < 0.01; Figure 1) when adjusted by pen (*P* = 0.03). Predicted parasite burden of TSc = 0 (no ticks/ear) at wk 2, was 0.2% (95% CI: 0.1–0.3) for CON and 17.9% (95% CI: 14.8–21.0) for TT, and at wk 3 postintervention was 0.1% (95% CI: 0.0–0.2) for CON and 83.4% (95% CI: 75.9–88.9) for TT. The observed numbers and proportions of cows in each TSc category by week are presented in Supplemental Table S1 (see Notes). At wk 0, the observed proportions had been 0.3% and 1.2%, respectively. However, no significant effect of treatment and treatment × week interaction was observed for weekly milk yield (*P* = 0.52; Figure 2). The overall mean milk yield for CON and TT was 39.7 (95% CI: 39.5–39.9) and 39.6 (95% CI: 39.4–39.8) kg/d, respectively, when adjusted by pen (*P* = 0.42).

**Figure 1:**
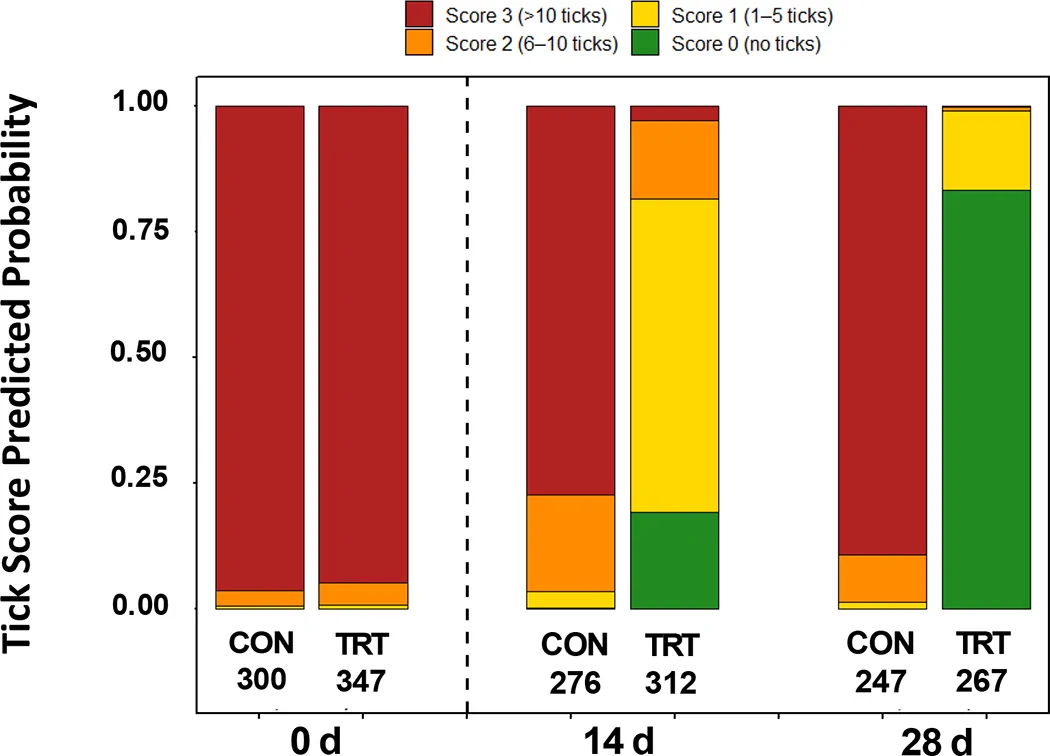
Spinose ear tick score predictive probability for Holstein cows assigned to TT (∼3 mL of 0.50% permethrin aerosol spray applied in ears at 0 and 2 wk; n = 347) or CON (no intervention; n = 300) in a California freestall dairy. At enrollment (baseline = wk 0), cows in each treatment group had similar tick scores (*P* = 0.39). Significant effects were observed for treatment effect (*P* < 0.001) and treatment × week (*P* < 0.001) when adjusting for pen (*P* = 0.03).

**Figure 2:**
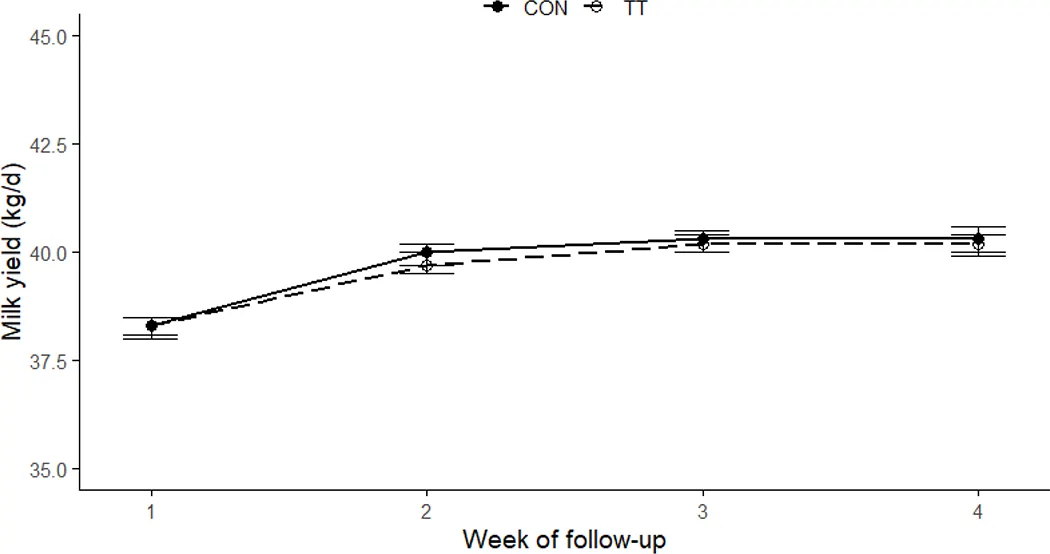
Weekly milk yield (LSM ± SE) of Holstein cows assigned to either a treated group (TT; ∼3 mL of 0.50% permethrin aerosol spray applied in ears at 0 and 2 wk; n = 347) or a no-intervention control group (CON; n = 300). When adjusting for baseline milk yield (*P* < 0.001) and pen (*P* = 0.42), no significant effects of treatment (*P* = 0.52) or treatment × week interaction were detected (*P* = 0.52).

Spinose ear tick infestation is an emerging concern for California dairy producers, yet evidence regarding effective control strategies and their effect on dairy performance under commercial conditions remains scarce. This field trial contributes new information by evaluating both tick burden and short-term milk yield following topical permethrin treatment applied in the ears of cattle in a commercial dairy herd. Although 2 applications of a 0.5% permethrin aerosol markedly reduced tick burden, this reduction did not translate into detectable changes in milk yield during the 28-d follow-up period.

Topical insecticides, delivered as sprays or impregnated ear tags, have previously demonstrated the ability to reduce *Otobius megnini* burdens (Kemper and Peterson, 1953; Drummond et al., 1967; Lancaster, 1984), although residual efficacy often diminishes within 1 mo. Conversely, systemic endectocides, whether injectable (ivermectin, doramectin, eprinomectin) or pour-on formulations (sulfur, famphur, eprinomectin) have consistently performed poorly (Campbell and Benz, 1984; Facchin et al., 1998; Nava and Guglielmone, 2009). This is likely because therapeutic concentrations do not reach the deep ear canal where larvae and nymphs feed for extended periods. For these reasons, a direct in-ear topical approach was selected for this study. Aerosol spray was chosen over impregnated ear tags to avoid potential insecticide transfer between TT and control CON cows housed in close proximity.

Several factors may explain the lack of a milk-yield response. First, the production impact of spinose ear tick infestation may be modest under the conditions of this herd. Second, any production benefits could have been delayed beyond the 4-wk follow-up, given that TSc were still declining at wk 3. At enrollment, 71% of cows were classified as TSc = 3, indicating substantial infestation. To determine whether heavily infested cows responded differently, a subgroup analysis restricted to cows with TSc = 3 was conducted. Results were similar to those of the full dataset, suggesting that initial parasite burden alone did not influence the milk-yield response to treatment.

Economic and welfare implications of spinose ear tick infestation have largely been inferred from observational reports rather than controlled trials (Strickland et al., 1976; Bulman and Walker, 1979). Severe infestations can lead to occlusion of the ear canal with wax, ticks, and debris; ulceration; and auricular nerve irritation, accompanied by head shaking, ear flapping, and rubbing that may interfere with feeding, rest, and social interactions (Rich, 1957). Historical accounts even link heavy infestations with mortality in cattle (Rich, 1957). In the present study, debris accumulation within the ear canal was a frequent gross finding, particularly in heavily infested cows; however, these changes were not systematically recorded, precluding formal assessment of lesion type, severity, or prevalence. Future studies should incorporate a standardized ear pathology and welfare scoring system to better quantify the clinical effect of spinose ear tick infestation and its potential relationship with production outcomes. Although no short-term milk-yield response was detected in this trial, the producer reported reduced ear tag loss at the end of the study. After study completion, the producer implemented a whole-herd control program using permethrin-impregnated ear tags and observed further reductions in tag loss. The dairy producer also perceived an improved BCS.

Managing spinose ear tick is inherently challenging because of its 1-host life cycle. Only larvae and nymphs parasitize cattle and reside deep in the ear canal for months, whereas nonfeeding adults live off the host and may lay multiple egg batches over an extended period. In freestall systems, engorged nymphs often drop into bedding where cows rest their heads, creating protected environmental reservoirs that sustain reinfestation (Hooker et al., 1912; Bulman and Walker, 1979). The aerosol permethrin protocol used here targets the ear canal effectively under conditions of high reinfestation pressure, but it requires intensive labor, close restraint of animals, and attention to handler safety, which limits scalability in large operations. Permethrin-impregnated ear tags offer a more practical long-term alternative by providing sustained release of insecticide (Lancaster, 1984). Although tags were intentionally avoided during the trial to maintain separation between TT and CON cows, they were adopted herd-wide after the study.

Tick scores increased in CON cows during the trial (the proportion with TSc = 3 rose from 83.6% at wk 2 to 90.8% at wk 3). The cause of this rise is uncertain, but a temporal increase in tick abundance over the study period is a plausible explanation.

Spinose ear ticks can harbor pathogens such as *Coxiella burnetii* and *Babesia* spp., although their vector capacity appears to be limited by their biology (Jellison et al., 1948; Diyes et al., 2018). In regions where New World screwworm (*Cochliomyia hominivorax*) is present or expanding, tick-induced ear lesions may predispose cattle to myiasis (Parish, 1942; Kemper and Peterson, 1953). As screwworm advances northward through Central America and Mexico (USDA–APHIS, 2024), maintaining effective tick control could become increasingly important for both welfare and biosecurity.

This study had several limitations. Treatment allocation using odd and even ear tags was not blinded, although tick assessments were performed by 1 team member while a separate team member recorded the cow ear tag number and associated TSc. This approach was chosen to minimize handling under commercial conditions and reduce the overall lock-up time for each cattle pen. In addition, although the study pens were initially selected to maintain stable cow groups throughout the trial, the producer was ultimately unable to keep all cows in the same pens because of routine herd management needs, as reflected by the cows lost to follow-up. The follow-up period for TSc (3 wk) and milk yield (4 wk) was also limited because, after completion of the trial, the producer elected to proceed with a whole-herd spinose ear tick eradication program. Despite these constraints, treatment groups were well balanced at baseline for parity, milk yield, and TSc, and losses to follow-up were comparable between groups.

In conclusion, 2 applications of 0.5% permethrin aerosol substantially reduced spinose ear tick burden in this commercial California dairy herd but did not improve short-term milk yield. The lack of a milk production response may reflect persistent ear pathology, the short observation period, individual variability among cows, or a limited overall effect of infestation on milk yield under these conditions. Future studies should prioritize evaluation, extend follow-up, and quantify both welfare and economic outcomes to clarify whether effective tick control yields measurable benefits for dairy operations.
